# Nesting the SIRV model with NAR, LSTM and statistical methods to fit and predict COVID-19 epidemic trend in Africa

**DOI:** 10.1186/s12889-023-14992-6

**Published:** 2023-01-19

**Authors:** Xu-Dong Liu, Wei Wang, Yi Yang, Bo-Han Hou, Toba Stephen Olasehinde, Ning Feng, Xiao-Ping Dong

**Affiliations:** 1 Faculty of Information Technology, Beijing University of Technology, Chaoyang District, Beijing, 100124 P. R. China; 2 Key Laboratory of Computational Intelligence and Intelligent Systems, Beijing University of Technology, Chaoyang District, Beijing, 100124 P. R. China; 3 Institute of Agricultural Economics and Development, Graduate School of Chinese Academy of Agricultural Sciences, 12 Zhongguancun South Street, Haidian District, Beijing, 100098 P. R. China; 4 Center for Global Public Health, Chinese Center for Disease Control and Prevention, Room 211, 155 Changbai Road, Changping District, Beijing, 102206 P. R. China; 5 National Institute for Viral Disease Control and Prevention, Chinese Center for Disease Control and Prevention, 155 Changbai Road, Changping District, 102206 Beijing, China

**Keywords:** COVID-19, Nested model, Functionalized β, Machine learning, ARIMA, SIRV model, Epidemic

## Abstract

**Objective:**

Compared with other regions in the world, the transmission characteristics of the COVID-19 epidemic in Africa are more obvious, has a unique transmission mode in this region; At the same time, the data related to the COVID-19 epidemic in Africa is characterized by low data quality and incomplete data coverage, which makes the prediction method of COVID-19 epidemic suitable for other regions unable to achieve good results in Africa. In order to solve the above problems, this paper proposes a prediction method that nests the in-depth learning method in the mechanism model. From the experimental results, it can better solve the above problems and better adapt to the transmission characteristics of the COVID-19 epidemic in African countries.

**Methods:**

Based on the SIRV model, the COVID-19 transmission rate and trend from September 2021 to January 2022 of the top 15 African countries (South Africa, Morocco, Tunisia, Libya, Egypt, Ethiopia, Kenya, Zambia, Algeria, Botswana, Nigeria, Zimbabwe, Mozambique, Uganda, and Ghana) in the accumulative number of COVID-19 confirmed cases was fitted by using the data from Worldometer. Non-autoregressive (NAR), Long-short term memory (LSTM), Autoregressive integrated moving average (ARIMA) models, Gaussian and polynomial functions were used to predict the transmission rate β in the next 7, 14, and 21 days. Then, the predicted transmission rate βs were substituted into the SIRV model to predict the number of the COVID-19 active cases. The error analysis was conducted using root-mean-square error (RMSE) and mean absolute percentage error (MAPE).

**Results:**

The fitting curves of the 7, 14, and 21 days were consistent with and higher than the original curves of daily active cases (DAC). The MAPE between the fitted and original 7-day DAC was only 1.15% and increased with the longer of predict days. Both the predicted β and DAC of the next 7, 14, and 21 days by NAR and LSTM nested models were closer to the real ones than other three ones. The minimum RMSEs for the predicted number of COVID-19 active cases in the next 7, 14, and 21 days were 12,974, 14,152, and 12,211 people, respectively when the order of magnitude for was 10^6^, with the minimum MAPE being 1.79%, 1.97%, and 1.64%, respectively.

**Conclusion:**

Nesting the SIRV model with NAR, LSTM, ARIMA methods etc. through functionalizing β respectively could obtain more accurate fitting and predicting results than these models/methods alone for the number of confirmed COVID-19 cases in Africa in which nesting with NAR had the highest accuracy for the 14-day and 21-day predictions. The nested model was of high significance for early understanding of the COVID-19 disease burden and preparedness for the response.

## Introduction

Since the outbreak of COVID-19, many scholars have used statistical methods [1–5], mechanism models [6–10] and deep learning methods [11–18] to predict the COVID-19 epidemic trend. Although the pure deep learning method has higher accuracy when compared with mechanism model in predicting the development trend of the epidemic, it cannot reflect the specific influencing factors of the epidemic trend like mechanism model and is less instructive for further taking corresponding prevention and control measures. So, the authors are attempting to combine the mechanism model with the deep learning method to obtain the advantages of both, that can not only provide more accurate analysis on the development trend of the epidemic but also analyze the influencing factors of the development of the epidemic and provide clues for further prevention and control.

Thus, our focus of interest was put to the functionalization of transmission rate β. A few of researchers have redefined the population classification based on the SIR model in order to predict the epidemic trend of COVID-19 more accurately [6–10]. These methods, however, ignored the time-varying characteristics of the transmission rate β so that they were not capable to improve the prediction accuracy of COVID-19 effectively. Some researchers discretized β, added asymptomatic infected persons and other factors such as social distance to SIR model to improve prediction accuracy [19]. Similarly, Deepa [20] functionalized the β staged with exponential function by adding the vaccine growth rate to the model to predict the end time of the epidemic in the United States, India, and Brazil. Zhang [21] et al. used genetic algorithms to optimize transmission rate and used a SIQR model to predict confirmed cases and deaths in Brazil. Zeng et al. [22] studied the effect of the government interventions as a condition for β function, argued that β was constant without government interventions, and became an exponential function after the government interventions, and the rate of recovery was an exponential power function by case study about the COVID-19 epidemic data of Hubei province, China, France, and the United States. Ding et al. [23] used the least-squares method to estimate β under different blockade states in South Africa using the Eureqa software [24] to obtain long-term predictions of daily growth rate, cumulative clearance rate, and cumulative mortality, and predicted the inflection point occurrence time. Kian et al. [25] used β daily decay function and added the ratio of depletion to the β function to simulate the early spread of COVID-19.

Machine learning and statistical methods can make relatively accurate short-term predictions, but they have higher requirements on training data. The prediction results are only related to the training data. Ardabili et al. [26] used genetic algorithms, particle swarm optimization algorithms, and gray wolf optimizer to estimate parameters in prediction models such as power functions, and used two machine learning algorithms to directly predict the infected cases, and the prediction results obtained were more accurate than prediction models such as power functions. However, the SIR model can predict long-term trends while the data requirements are not high. Yang et al. [27] added migrants on the basis of SEIR, compared the number of infected cases under the same control measures at different times in Wuhan, and used the LSTM method that used the number of SARS infections in 2003 as training data to predict the cases of COVID-19 infect. Therefore, other factors such as machine learning methods can be introduced to expand the model.

To this end, the authors established a nested model to incorporate machine learning methods including Non-autoregressive (NAR) and Long-short term memory (LSTM) models respectively in the mechanism model (SIR model) to fit the β. Besides, statistical methods including Autoregressive integrated moving average (ARIMA) model and Gaussian function as well as polynomial function etc. were also tried respectively to functionalize β. In addition, these methods have analyzed the factors that could influence the development of the epidemic and provide clues for further prevention and control.

Given the rapid spread of COVID-19 and the relatively low capacity of African countries to deal with the health situations, African countries have become more vulnerable. Hence, it is necessary to predict the number of the COVID-19 active cases, and the prediction results can help African countries minimize the losses.

Concretely, this study analyzed the development trend of the COVID-19 epidemic in the top 15 African countries (South Africa, Morocco, Tunisia, Libya, Egypt, Ethiopia, Kenya, Zambia, Algeria, Botswana, Nigeria, Zimbabwe, Mozambique, Uganda, and Ghana) with the cumulative number of active cases and discussed the predictability of the epidemic using the nested model stated above. On July 6, 2021, the African Center for Disease Control and Prevention issued a guideline for the simulation and prediction methods of the COVID-19 pneumonia epidemic in Africa [28], given its imperfections that neither the latest experienced different variant strains such as Delta and Omicron variants with different R_0_s of the epidemic nor vaccination measures in African countries has yet been covered. Realistically speaking, the epidemic statistics of African countries have low data density and abnormal data. For example, the number of vaccinations for several consecutive days may remain unchanged. The vaccine data for several consecutive days maybe vacant. The vaccine data suddenly increases, and the number of confirmed cases drops rapidly for unknown reasons and then rises rapidly to a higher position.

The combined nested SIRV model can predict the transmission rate in African countries with low data density and outliers using machine learning and statistical methods. With this model, the number of active cases can be more accurately predicted.

Accordingly, our nested model introduced a population “V” who has completed the whole process of vaccination against COVID-19 to form a SIRV model. Then, we obtained the transmission rate β of 15 African countries and predicted the daily β value with time-variable characteristics using five methods including NAR, LSTM, ARIMA, Gaussian function and polynomial function. As we know, the transmission rate β determines the change in the number of active cases in the traditional SIR model. After being functionalized, the β was substituted into the mechanism model to get the predicted value of the number of active cases. The nested model incorporating machine learning in SIRV model designed in this study has entirely considered the impact of the vaccination rate, effective time, and efficacy rate of the COVID-19 vaccine on the spread of the COVID-19 epidemic. We provided an expanded model involving the vaccine type and variant strains of the COVID-19 virus. Meanwhile, we have also compared the difference in the prediction accuracy of infection numbers between machine learning, statistical methods alone and their nested models respectively.

## Methods

### Data sources

All data used in this study are from Worldometer [29]. The duration of the data was from September 2021 to January 2022. The Government Policy Relevance Index comes from the COVID-19 Government Response Tracker (OxCGRT) developed by Oxford University.

### The Construction of nested model

**Fig. 1:**
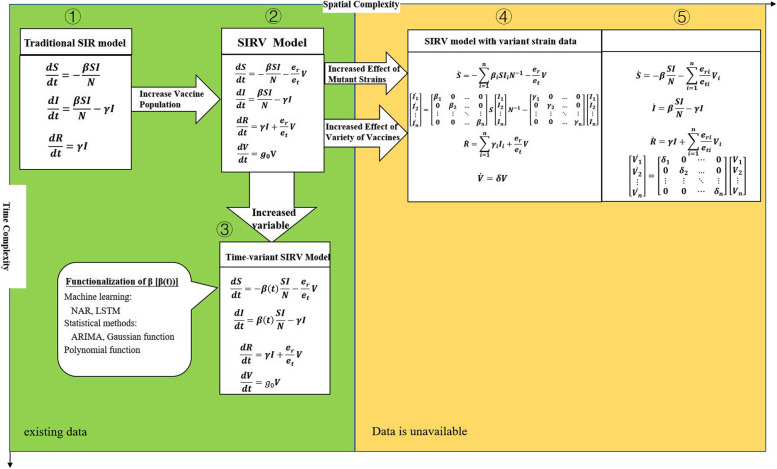
The designed procedure for nesting machine learning and statistical methods in SIRV model

The construction of the nested model incorporating machine learning and statistical methods in SIRV model proposed in this study began with the SIR model. After adding the time-varying data of the whole process of vaccination against COVID-19, SIRV model was obtained, and then the dynamically changing transmission rate β in sequence (③ in Fig. 1, Table 1). The e_r_ was set as 0.7, and e_t_ represented the vaccine effective time which was set as 14 days in this model (e_r_, e_t_ are based on expert experience). Ideally, the model shown in ④ (Fig. 1) could be obtained by considering the data about variant strains such as the transmission rate β, removal rate γ, and the number of infected cases “I” (active cases) of different variant strains. These indices were expanded to the transmission rates β_1_, β_2_……β_n_, the removal rates γ_1_, γ_2_……γ_n_, and the number of infected cases I_1_, I_2_……I_n_. Similarly, if different vaccine types were considered based on ②, it can be extended to shown in ⑤. While, both of models ④ and ⑤ were lack of data in reality to construct our nested model further (Fig. 1).

**Table 1:** The meaning and of each letter in the part ③ of the differential equation

**Variable**	**Meaning**	**Whether a time variable**
$\mathrm{S}$	Susceptible	Yes
I	Infected	Yes
$\mathrm{R}$	Removed	Yes
$\mathrm{V}$	The COVID-19 Vaccine	Yes
$\mathrm{N}$	Total Population of Each Country	No
β	Transmission rate	Yes
${e}_{r}$	Vaccine Efficacy	No
${e}_{t}$	Vaccine Effective Date	No
γ	Removal rate	No
${g}_{0}$	Vaccination Growth Rate	No

**Fig. 2:**
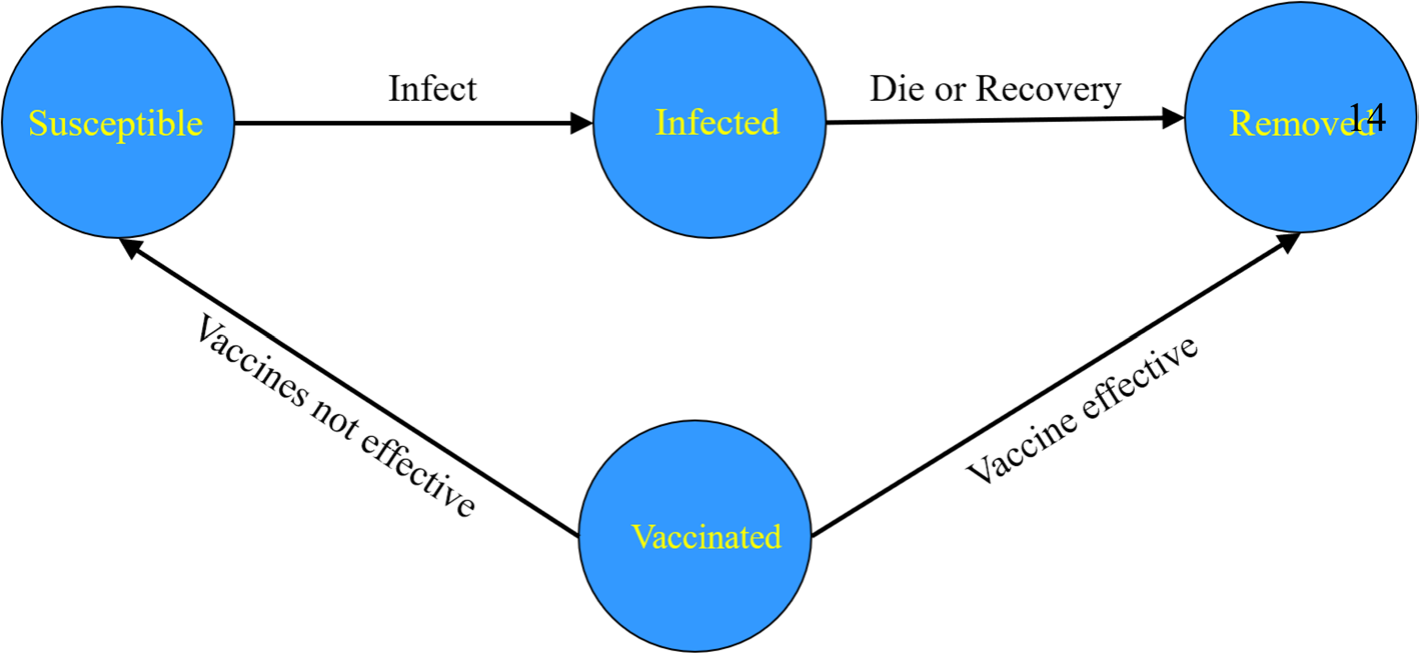
The interaction among S, I, R and V

For the SIRV model in our nested model, we categorized unvaccinated people, the people who are not infected with the COVID-19, and the people who have received the COVID-19 vaccine but have not yet taken effect into susceptible groups i.e. “S”. The people infected with the COVID-19 were classified as the infected population “I”, and the people who recovered after being infected with the COVID-19, those who died from the infection and the effective COVID-19 vaccine were classified as the removed population, that is, the population “R” who did not infect or spread the COVID-19 virus. The interaction of these variables is shown in Fig. 2. In this study, the effective population was defined as people who have been vaccinated with all doses of COVID-19 vaccine and have successfully established COVID-19 virus immunity; and those who have not been in effect are people who have not been fully vaccinated with COVID-19 vaccine or have received full doses of COVID-19 vaccine but have not successfully established COVID-19 virus immunity.

The four differential equations in the Part ③ of Fig. 1 are explained as follows. The first equation is the susceptibility rate equation with *S*(*t*) is the susceptible population at time *t*, the second and the third equations describe the infection and removal rate respectively, with *I*(*t*) and *R*(*t*) are the infected and removed population at time *t*. The fourth equation describes people who has completed the whole process of vaccination against COVID-19 at time t. In these equations, *β* is the transmission rate, e_r_ is vaccine efficacy, *e*_*t*_ is vaccine effective date, *γ* is the removal rate which based on the expert experience collected by the authors and relevant actual data, the authors found that the average recovery time of the COVID-19 patients was 14 days, so the model established in this paper set the removal rate at 1/14, *g*_*0*_ is vaccination growth rate, and *N* is the total population in any region (Table 1). Here, we are assuming that the new births and deaths due to ageing, accidents, non-epidemic diseases, etc. are negligible. Then the total population *N* is always constant, so we have

$${\varvec{S}}\left({\varvec{t}}\right)+{\varvec{I}}\left({\varvec{t}}\right)+{\varvec{R}}\left({\varvec{t}}\right)={\varvec{N}}$$

$$\frac{dS}{dt}+\frac{dI}{dt}+\frac{dR}{dt}=0$$

Based on the second part of Fig. 1, the model can be extended to Equations shown in the ④ part of Fig. 1, by incorporating data on mutant strains.

After incorporating data on vaccine types, the model can be extended to Equations shown in the ⑤ part of Fig. 1.

### Model solution

**Fig. 3:**
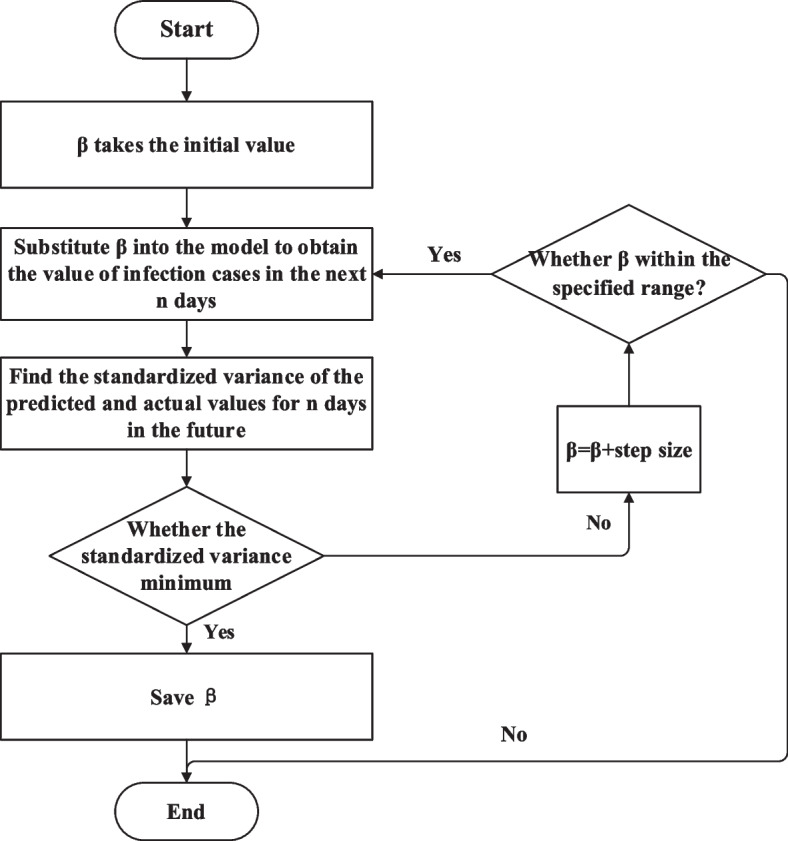
Traversing process of β on a certain day

Firstly, the validity period of the vaccine and the vaccine’s effectiveness were adjusted adaptively, and the time-varying transmission rate β was obtained through traversal. Then β was calculated by five methods, including NAR, LSTM, ARIMA, Gaussian function and polynomial function. After fitting and prediction, the obtained β was finally substituted into the SIRV model to obtain the predicted value of the number of infected cases. The traversing process of the β value for one of the days is shown in Fig. 3.

**Fig. 4:**
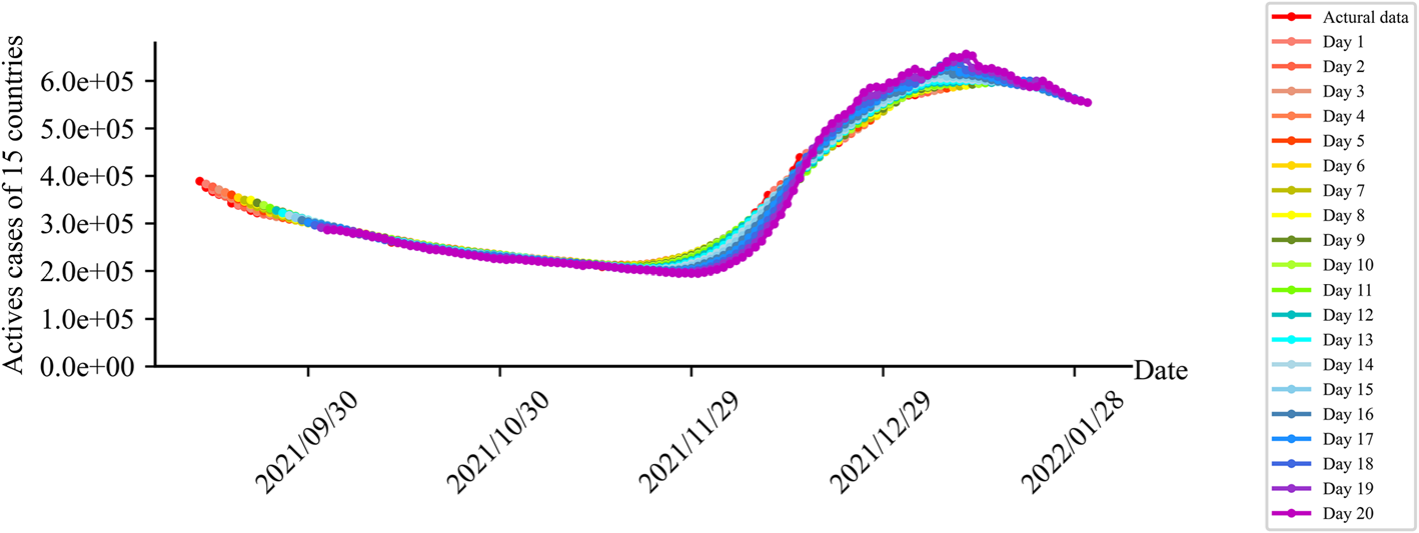
Fitting results of the number of active cases of COVID-19 in 15 African countries

Secondly, the obtained β was substituted into the SIRV model to fit the epidemic trends of 7, 14, and 21 days of the 15 African countries (Fig. 4). Mean absolute percentage errors (MAPEs) of the comparisons between the simulations of 7, 14, and 21 days and the real epidemic trends were 1.15%, 2.41%, and 4.61%, respectively. The simulated trend was consistent with the real epidemic trend, so this model can be considered valid.

### β prediction methods

In this paper, the β value is a factor that describes the intensity of the virus infection, which can be calculated using the SIRV model described in this article from the number of existing infected people. The specific calculation method is to use the traversal method to traverse all possible β values in days within a reasonable interval, and predict the number of cases in the next 7 days according to the β values obtained by all traversals, and finally select the β value with the smallest error as the β value in the dataset. At the same time, the LSTM, ARIMA and other methods used in this paper are also trained according to the β values obtained by the above described methods as datasets, so as to obtain the β values in the future, and then use the β values obtained by training to substitute into the SIRV model again to obtain the predicted number of infected people and other data.

The experiments of β fitting and prediction were carried out from the easier methods to more difficult ones. So, the order was: polynomial function, Gaussian function, ARIMA, NAR and LSTM.

Firstly, a polynomial function was used. To avoid data over fitting, we repeated the experiments using a third-order polynomial function to fit β.

After traversing to obtain β, we found that it aligned more with the superposition state of multiple normal distributions within a certain period. Therefore, secondly, Gaussian function was used to fit the change process of β.

Thirdly, since the transmission rate β involved in this paper is a non-stationary time series, ARIMA was used to predict β.

Fourthly, due to the complex time-variable characteristics after traversal, it was difficult for ordinary linear methods to fit the transmission rate β.While NAR has feedback and memory capabilities, and its output at each moment correlates with both the input at the current moment, and the input and output at the last time. At the same time, NAR can perform dynamic calibration, which requires less time for model calibration; when new samples are added, the matrix order remains unchanged, and matrix inversion operations are not necessary. Therefore, the NAR method can improve computational efficiency so that it was used to fit the time variables.

Fifthly, compared with RNN, the output of each step of LSTM is not determined by the input of the last moment completely. Due to the structure of the forget gate, LSTM can perform a relatively reasonable and accurate long-term operation to predict sequence variables with a small computational cost. Therefore, this study also tried to use the LSTM method to fit the transmission rate β.

The beginning of September 2021 was set as the starting point of the training set. At that time, the 15 African countries studied in this paper had all launched the vaccination against the COVID-19. After this time point, Delta and Omicron variant strains gradually became the mainstream COVID-19 virus strains in Africa in succession.

### Policy-related indices

This study correlated the policy-related indices (from Oxford University COVID-19 Government Response Tracker) with the predicted quantity β to determine whether the use of government policy-related indices had a positive impact on the model prediction.

Spearman analysis method was used to analyze the rank correlation because the correlation was not linear and the data had a high degree of dispersion and abnormal points. The rank correlation between the β and traditional strictness index, government response index, containment correlation index, and economic support index were 0.105, -0.591, -0.560, and 0.378 respectively. There was no apparent correlation between β and each policy index, so the government policy correlation coefficient was defined as a reference variable in the model constructed in this study and was not directly involved in the actual operation of the model.

### Validation of the model

The validation of the model used datasets from two countries, Germany and the Netherlands. Because, they both rank among top 15 countries in the Human Development Index around the world, their reported cumulative numbers of COVID-19 cases were of the same order of magnitude with that of the 15 African countries we analyzed, and had a weak correlation between the government policy-related index and β that was similar to the 15 African countries.

## Result

### Fitness of the number of active cases

**Fig. 5:**
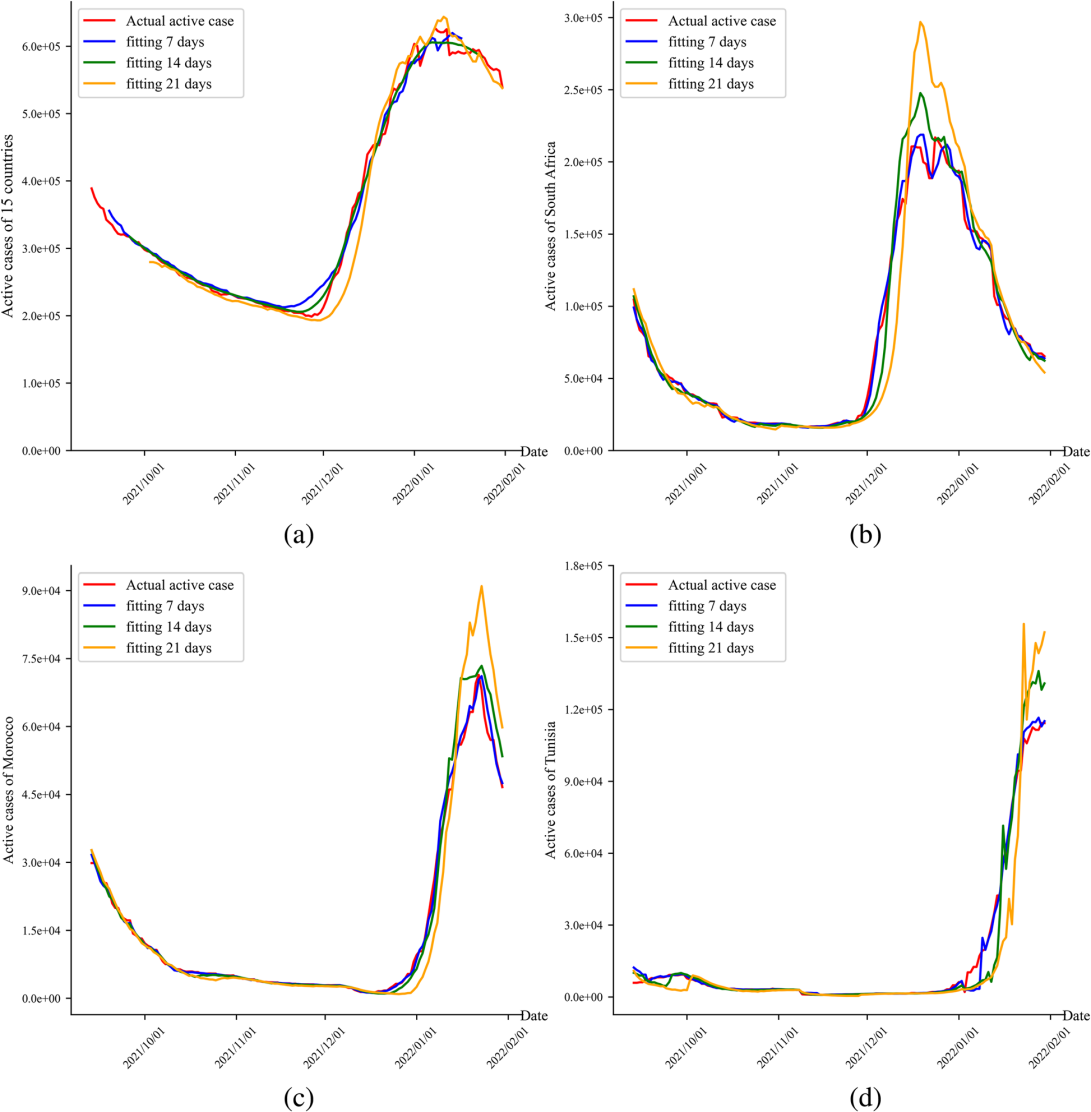
The fitting of the number of active cases for 7, 14 and 21 days. Note: **a** is the number of daily active cases in 15 countries, (**b**) is the number of daily active cases in South Africa, (**c**) is Morocco Number of daily active cases, and (**d**) is the number of daily active cases in Tunisia

The fitting curves of the 7, 14, and 21 days were consistent with the original curves of the original number of daily active cases (Fig. 5). The fitting errors increased from 1.15% gradually when the model fitted 7, 14 and 21 days. The fitted data of 7, 14 and 21 days were higher than the true values at the peak due to the systematic error at each model iteration.

### Prediction of transmission rate

**Fig. 6:**
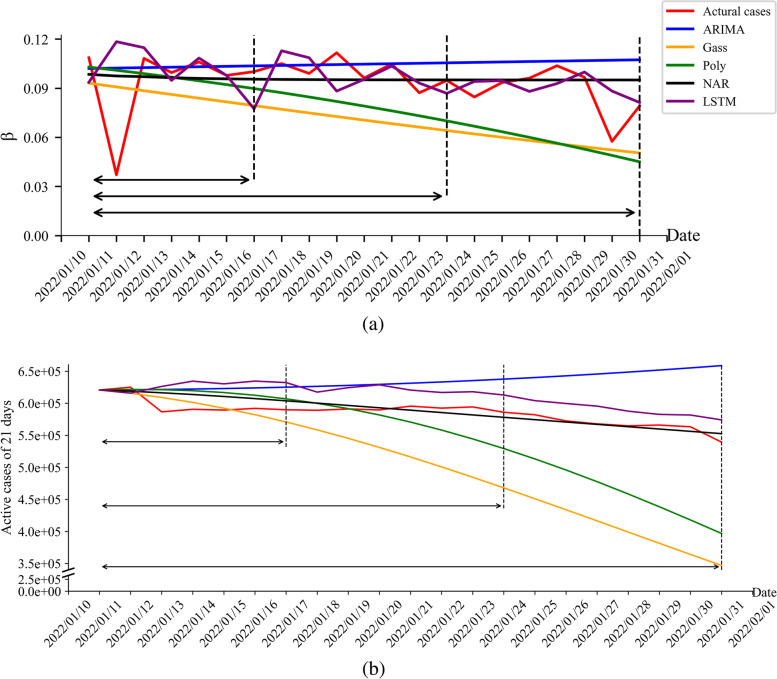
Comparisons on β and the number of active cases between the real and predicted data. Note: **a** Comparison between the real and predicted transmission rate β for 7, 14 and 21 days; **b** Comparison between the real and predicted numbers of active cases in the next 7, 14 and 21 days

The actual β fluctuates greatly in the first two days from the start of the prediction on January 11, 2022, and then the image oscillated in a “wavy” shape, showing an overall downward trend. The β predicted by LSTM fluctuated greatly from January 11, 2022 to January 17, 2022. Especially, the β value reached the highest value of 0.1184 on January 12, 2022, and then oscillated around the real β. The β obtained by the NAR method reached the highest point of 0.09843 on the first day, and then decreased monotonically and slowly. The β obtained by the ARIMA method increased smoothly and monotonically within the 21 days of prediction time range, and was always greater than 0.1. The β predicted by the Gaussian function was a monotonically decreasing curve, dropped from around 0.09 to 0.05. It had the most obvious downward trend among the five methods from January 11, 2022 to January 28, 2022.The β predicted by the polynomial function was consistent with the one by the Gaussian function, and both were monotonically decreasing curves. The downward trend of the β predicted by polynomial function was the highest among the five methods from January 29, 2022 to January 31, 2022 (Fig. 6a).

The prediction curve obtained by LSTM method had the largest fluctuation, and oscillated and decreased after reaching the maximum value. The NAR drops from the highest point to about 0.09, but it was obvious that the NAR method reached a stable statefirstly. The prediction curves obtained by statistical methods are all monotonic curves, the ARIMA prediction curve was a monotonically increasing curve, while the Gaussian function was monotonically decreasing curves with obvious downward trend. The β curve predicted by the polynomial function increased monotonically in the first two days, and then decreased monotonically (Fig. 6a).

**Fig. 7:**
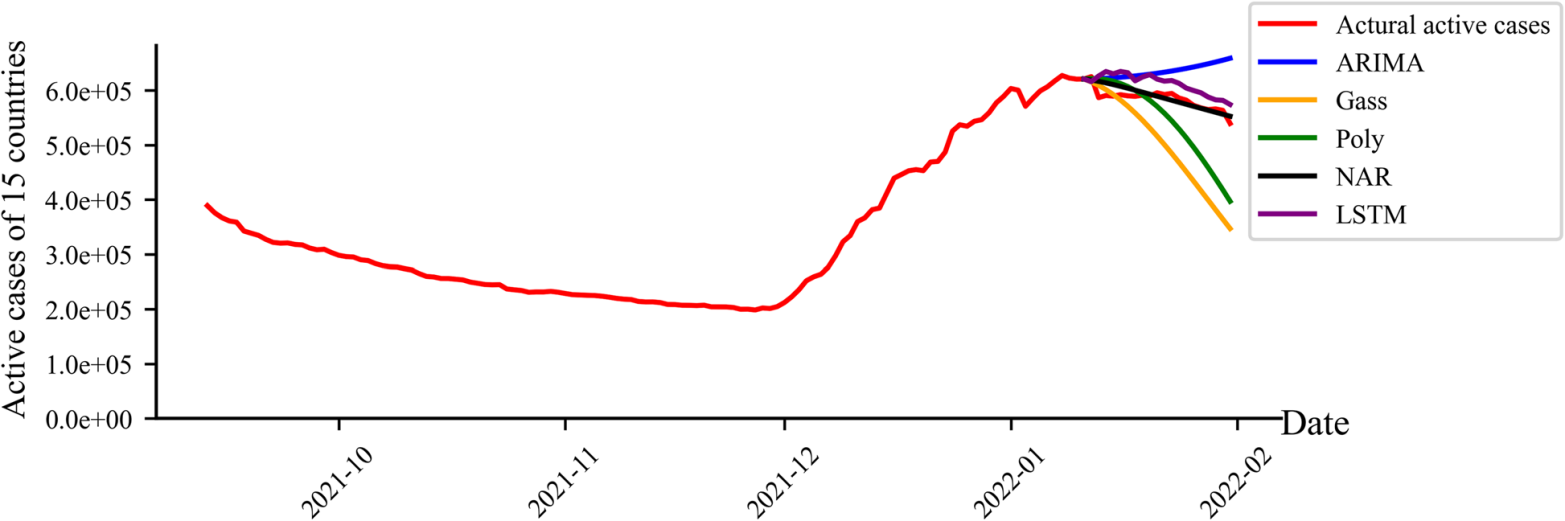
Comparison between the predicted inflection point and the real value

The true number of infection cases peaked on the second day, dropped sharply on the third day, and then fell in a “wave”. The number of active cases obtained by the LSTM method generally showed an upward trend in the first 10 days, and then decreased monotonically. The number of active cases obtained by the 21-day curve was always larger than the actual number of active cases; the number of active cases predicted by the NAR method was a monotonically decreasing curve. After the 10th day, the number of infections is always smaller than the true number of infections. The numbers of active cases predicted by the ARIMA method were always larger than the reported number of active cases from the third day, which was a monotonically increasing curve. For the Gaussian function, the predicted curve was a monotonically decreasing one with a decreasing slope, and the predicted number was smaller than the reported one on the sixth day. The predicted numbers of active cases by the polynomial function reached the maximum value on the second day, followed by a monotonically decreasing curve which was completely smaller than the reported number of active cases from the 10^th^ day (Fig. 6b). The predicted numbers of active cases by the machine learning methods were closer to the reported ones than those predicted by statistical and polynomial methods. The number of active cases predicted by the LSTM method and polynomial function increased firstly and decreased then, which could predict the peak of infection (Fig. 7).

**Fig. 8:**
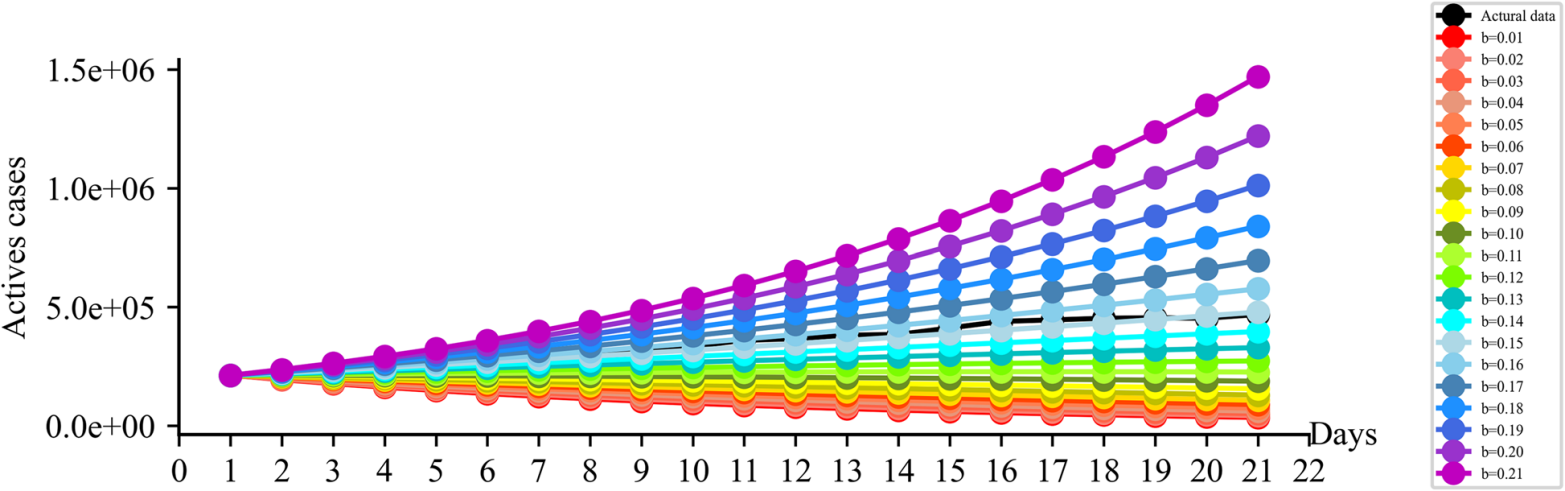
Different β corresponding to different sensitivities in 15 countries

When the transmission rate was smaller than the removal rate, and the smaller the transmission rate was, the lower the predicted number of active cases would be, so when the transmission rate was smaller or larger, the sensitivity was higher. In this study, β was taken from 0.01 to 0.21, and the removal rate was set to 0.1. The obtained curve of the number of active cases increased with the increasing of β. After the mathematical derivation of the original model and the analysis of the actual experimental results, it resulted in that the sensitivity of the nested model constructed in our study was positively correlated with β. Therefore, the predicted β and the number of active cases in our nested model were usually higher than the actual and reported ones slightly and respectively (Fig. 8).

### Comparison of prediction accuracy among the methods used

The comparison of the nested models incorporating with the 5 different methods in the SIRV method with the 5 different methods alone for the prediction of the development of the COVID-19 epidemic in Africa showed that the nested models had higher prediction accuracies than all of the 5 methods alone respectively.

**Fig. 9:**
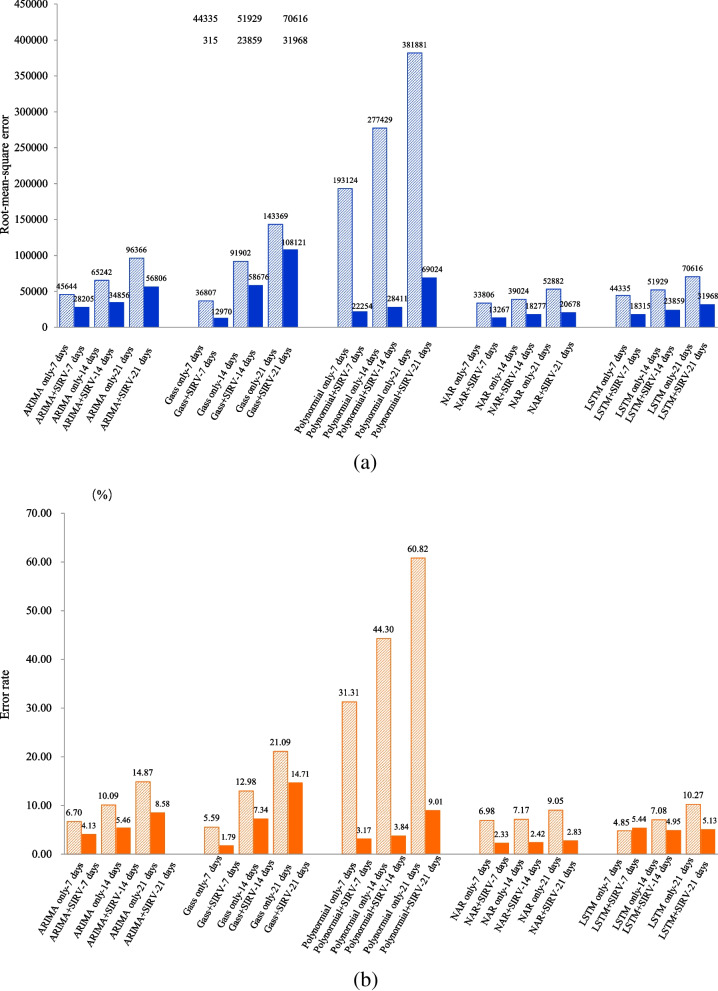
Comparison of RMSE and MAPE among each method nested in the SIRV model. Note: **a** showed the comparison of RMSE among each method, (**b**) showed the comparison of MAPE among each method

Among the 5 methods, the polynomial fitting method in the nested model has the most obvious improvement compared to using the polynomial fitting method alone in prediction, and the difference in MAPE was 51.86%. The RMSEs of using only polynomial method alone to predict the numbers of active cases were the largest in 7, 14, and 21 days, which were 193,124, 277,429, and 381,881 respectively. The RMSEs obtained by Gaussian function in the nested model was the smallest in the 7-day prediction (12,974). The RMSE/MAPE of the number of active cases predicted by NAR in the nested model was the smallest in 14 and 21 days, and the RMSEs/MAPEs by LSTM in the nested model were larger than those of NAR in the nested model in 14 and 21 days, but they were less than those of both statistical methods and polynomial function combined in the nested model (Fig. 9).

### Validation of the model

The results of using the nested model to predict the number of active cases in Germany are as follows: all three nesting methods predict an upward trend in the number of active cases, and the 21st MAPE of the nested LSTM model method is only 2.82%, which is the best result. The prediction results of the SIRV nested model for active cases in the Netherlands also successfully predicted the upward trend of the number of active cases. The 21-day MAPE of the prediction results of the nested ARIMA model method was 12.24%, which was the best among the three. Africa’s 21-day MAPE was less than 10% for all three hybrid methods, while Germany’s 21-day MAPE using the ARIMA hybrid method was greater than 10%, and the Netherlands’ MAPE using all three methods was greater than 10% (Table 2).

**Table 2:** Comparison of MAPEs between nested and single method for Germany and The Netherlands

Country	Methods	7 days		14 days		21 days	
		Nested	Single	Nested	Single	Nested	Single
Germany	NAR	2.21	19.00	3.48	30.60	5.92	38.58
	LSTM	2.31	4.66	2.62	13.33	2.82	24.19
	ARIMA	8.19	12.37	17.41	24.11	26.69	34.88
The Netherlands	NAR	4.91	3.52	14.76	13.61	25.32	26.16
	LSTM	2.17	5.96	6.52	14.74	12.88	24.66
	ARIMA	2.33	3.96	6.94	11.91	12.24	21.06

## Discussion

In order to provide better insights into the predictability of the epidemic trend of COVID-19 in Africa, we employed existing data that have been published in authoritative sources, and explored to take the advantages of both machine learning method and mechanism model (SIRV model) which are expected to accurately predict the epidemic trends and provide clues for further control and preventive measures at the same time. It could be inferred that the data on the COVID-19 epidemic in Africa are characterized with low density, abnormal data, missing reports and so on, thus this appears to be a limitation for use of the raw data set. This study therefore, explored a nested model incorporating machine learning methods, statistical methods and polynomial function respectively in SIRV model to allow for perfect utilization of the available data on Africa COVID-19 epidemic.

The SIRV model in this study was generated from SIR model by considering the vaccination effect. The core essence of “nesting”/“nested” used in the study denotes the functionalization of transmission rate β in the SIRV model by using machine learning methods (NAR, LSTM), statistical methods (ARIMA, Gaussian function) and polynomial function respectively. As a result, the nested model created showed a satisfactory accuracy in the prediction of the epidemic trends of COVID-19 in African countries, especially for the SIRV model nested with machine learning methods. Even, when we used data of the Netherlands and Germany over the same period, the prediction results obtained by using the nested model were better than those obtained by applying only a single model, but the error was higher generally than that of African countries. Here, we will discuss the prediction result, accumulative error and sensitivity of the nested model constructed in this study, and the feasibility to combine the mechanism (SIRV) model with machine learning method for the prediction of trend epidemic of COVID-19.

As we know, machine learning method has higher prediction accuracy than statistical method and polynomial function when they are used alone. This study showed that the nested models incorporating machine learning methods in SIRV model also had higher prediction accuracy than those incorporated with statistical methods and polynomial function. At the same time, the two machine learning methods adopted were very suitable for the prediction of one-dimensional time series after being nested in SIRV model. One reason for this is the innate characteristics of machine learning method in that LSTM is derived from RNN model designed for time series specially and NAR is suitable for the prediction of one-dimensional time series in the presence of oscillation [30, 31]. The other reason is that the β value processed by the nested model was of irregular oscillation that was more suitable for both NAR and LSTM to predict than other methods. However, the parameters of the NAR dynamic neural network need to be manually adjusted, and the current parameters may not be applicable to the data of other countries, which limits the application of NAR in other practices [32].

Accordingly, as shown in Fig. 7 of the results section, the difference between the various curves can be explained by the memory cells of LSTM to store past data and the feedback mechanism of NAR, the weakness of ARIMA in processing irregular oscillation data, and that both the second-order Gaussian and the third-order polynomial function were showing monotonous decreasing when predicting the β respectively.

During the experimental process, we found that the nested model with polynomial function had the biggest improvement effect than others in the prediction of infected case number. The possible reason was that the accuracy of polynomial fitting correlated with the polynomial order used. Both under-fitting and over-fitting of polynomial function in direct prediction could cause huge deviations, and the higher the fitting order was, the larger the deviation. While, compared with the direct prediction of the number of active cases, the order of magnitude of the predicted β was smaller, so smaller was the corresponding fitting error.

The β obtained in this study could be substituted back to the nested model through two approaches to predict the number of active cases. One is to use the β predicted by the five methods to make a one-time prediction on the number of active cases of the next 7, 14 and 21 days. Another is that each of the time variable β is used to predict the number of active cases of only one day after the current day, then day by day for the next 7, 14 and 21 days. According to the experimental results, the latter approach could predict the number of active cases more accurately to reduce the cumulative error that occurs during the integration process.

Regarding the sensitivity of the model, the nested model incorporating machine learning etc. in SIRV model designed in this study had a different sensitivity to different infection situations (Fig. 8). The sensitivity of the nested model we constructed in this study correlated with β positively. When the transmission rate β is equally spaced, the number of active cases presents different sensitivity distributions. When β is higher than the removal rate, the sensitivity of the model rises significantly. Therefore, the predicted β and the number of active cases built in this study were usually slightly higher than the actual situation at the peak, the estimated number of active cases is generally pessimistic in the early stage of a round of infection. This method is not only very suitable for estimation before an outbreak of the epidemic but also can be sensitive to predict the inflection point of the outbreak, so as to provide more reliable short, medium- and long-term epidemic-related forecasting data forecasts to facilitate the development of pertaining control measures and policies. Comparing a direct use of machine learning or statistical methods to predict the number of active cases, we showed that the nested method could effectively estimate the outbreak degree of a wave of epidemics and correct the underestimation of the transmission rate from the algorithm level, which can play a specific warning role.

In the view of the nested model created in this study was based on the SIR epidemiological model, the final prediction accuracy was closely related to the specific transmission mode of the virus. For example, the removal rate was negatively correlated with the virulence of the variant strain, and the β was positively correlated with the infectivity of the variant strain. However, the changes in infectivity and pathogenicity caused by the mutation of the virus have a fundamental impact on the development trend of the epidemic, the virus variation has a critical impact on the transmission rate and removal rate, and the variation of the virus cannot be predicted accurately currently, so that our nested model was good at early warning of outbreak, and capable to predict the epidemic inflection point in the early and middle stages of a wave of epidemic, but not good at the prediction of the future epidemic if neither the new variation of the virus nor its characteristics of virulence or transmission are well mastered. The reason for the strength of our nested model is that, in the early stage of the epidemic, the β value will show an upward trend. When the β value rises above the outbreak threshold and is in the rising range, it will sharply increase the number of active cases. This situation corresponds to a large-scale outbreak that may be caused in the early stage of the epidemic.

Regarding R_0_, β and R_0_ belong to the same type of indicators. In the process of using machine learning and statistical methods to predict β, the calculation of R_0_ has been included, so R_0_ is not involved in this study.

In addition, because many of the top 15 African countries with cumulative confirmed cases lack the COVID-19 vaccination data, we used the hot platform imputation method to fill the missing values of vaccine data in this study. Through our ongoing further research, it has been found that there are still other methods available to fitting the missing data. We will continue to explore superior method to fill the missing values for future solutions.

## Conclusion

Nesting the SIRV model with NAR, LSTM, ARIMA methods, Gaussian and polynomial functions through functionalizing β respectively could obtain more accurate fitting and predicting results than these models/methods alone for the number of confirmed COVID-19 cases in Africa processing low data density, a few of outliers and missing reports. The nested SIRV model with NAR had the highest accuracy for the 14-day and 21-day predictions, while that with Gaussian function had the highest accuracy for 7-day prediction.

The prediction method in this paper is significant to the real-world COVID-19 response in many ways and manners. Namely, it can predict the local burden of the COVID-19 pandemic and thus can enhance the preparedness of the government for the pandemic response, including the early preparedness of supplies, technologies and resources.

## Data Availability

The datasets analysed during the current study are available in the [Worldometer] repository, [https://www.worldometers.info/coronavirus]. The duration of the data was from September 2021 to January 2022. The Government Policy Relevance Index comes from the COVID-19 Government Response Tracker (OxCGRT) developed by Oxford University.

## Abbreviations

ARIMA: Autoregressive integrated moving average
DAC: Daily active cases
LSTM: Long-short term memory
MAPE: Mean absolute percentage error
NAR: Non-autoregressive
RMSE: Root-mean-square error
